# Robust yet deformable nanodomains for super-strong and ultra-tough reversibly cross-linked plastics

**DOI:** 10.1093/nsr/nwaf512

**Published:** 2025-11-17

**Authors:** Chengliang Tao, Xingyuan Lu, Xiang Li, Junqi Sun

**Affiliations:** State Key Laboratory of Supramolecular Structure and Materials, College of Chemistry, Jilin University, Changchun 130012, China; State Key Laboratory of Supramolecular Structure and Materials, College of Chemistry, Jilin University, Changchun 130012, China; State Key Laboratory of Supramolecular Structure and Materials, College of Chemistry, Jilin University, Changchun 130012, China; State Key Laboratory of Supramolecular Structure and Materials, College of Chemistry, Jilin University, Changchun 130012, China

**Keywords:** reversibly cross-linked polymers, tough plastics, phase-separated nanodomains, carbon fiber-reinforced polymer composites, impact-resistant plastics

## Abstract

High-performance plastics that simultaneously integrate exceptional strength, modulus, toughness and recyclability remain elusive owing to intrinsic property trade-offs. Here, we report super-strong and ultra-tough reversibly cross-linked poly(aryl amide)–polyurea (PA–PU) plastics obtained by copolymerizing rigid PA and flexible PU chains. *In situ*-formed hydrogen-bonded PU nanodomains act as robust yet deformable cross-links, reinforcing strength of the plastics while efficiently dissipating energy through nanodomain deformation and hydrogen-bond dissociation. As a result, the PA–PU plastics exhibit a tensile strength of 103.7 MPa, a Young’s modulus of 2.5 GPa and a toughness of 36.1 MJ m^−3^, along with satisfactory thermal resistance, chemical stability and re-processability. Incorporation with carbon fibers (CFs) produces PA–PU/CF composites with superior tensile and flexural properties, outstanding low-temperature impact resistance and solvent-assisted recyclability, outperforming conventional epoxy/CF composites. This work establishes that *in situ*-formed nanodomains combining mechanical robustness with deformability offer an effective strategy to toughen plastics.

## Teaser

In situ-formed hydrogen-bonded, robust yet deformable nanodomains effectively reinforce reversibly cross-linked networks, yielding super-strong, ultra-tough plastics with excellent thermal resistance, chemical stability, and reprocessability.

## INTRODUCTION

The rapid development of the automotive, aerospace, aviation and biomedical industries has created an urgent demand for high-performance plastics that simultaneously offer superior mechanical strength, high modulus and excellent toughness [1–7]. These plastics can efficiently dissipate energy under external stress to preserve structural integrity and avoid brittle fracture, which is commonly observed in mechanically strong plastics with low toughness [2,6,8–11]. However, achieving exceptional strength, modulus and toughness in a single plastic remains a formidable challenge owing to the intrinsic trade-offs among these properties [3,12–14]. High strength and modulus typically require rigid structural motifs (e.g. aromatic rings) and high cross-linking densities, both of which strongly restrict chain mobility and suppress plastic deformation [3,15–20]. As a result, the plastic deformation zone around a crack tip, which is critical for dissipating local stress, is greatly reduced, leading to poor toughness. To overcome this dilemma, a widely adopted approach involves incorporating flexible secondary polymers into rigid plastics to create heterogeneously toughened plastics [6,8,21–26]. The thermodynamic immiscibility between rigid and flexible polymers often induces the formation of phase-separated nanodomains, which enhance energy dissipation and improve toughness [2,8,23,24]. However, this improvement in toughness is frequently accompanied by reduced strength and modulus. An alternative approach is to reinforce polymers of low strength but relatively high toughness with dense supramolecular cross-links and/or nanofillers, thereby enhancing strength, modulus and toughness simultaneously [14,27–33]. Nevertheless, owing to the intrinsically soft nature of such matrices, achieving outstanding mechanical robustness and toughness remains a considerable challenge. This challenge is further exacerbated by the urgent demand for mechanically robust, tough and sustainable plastics. The covalently cross-linked networks that endow these plastics with high mechanical strength also render them inherently non-reprocessable, posing serious obstacles to end-of-life recycling [19,29,34–39]. Therefore, the development of next-generation plastics that simultaneously achieve high strength, modulus, toughness and recyclability remains both essential and profoundly difficult.

We believe that the *in situ* formation of nanodomains offers a highly promising strategy for simultaneously strengthening and toughening plastics. To achieve effective reinforcement, two essential conditions must be satisfied: (i) the nanodomains must possess excellent energy dissipation capacity; and (ii) they must exhibit strong interfacial interactions with the surrounding polymer matrix. Conventional *in situ*-formed nanodomains rarely satisfy these criteria [6,8,24]. To address these shortcomings, we envisaged incorporating supramolecular interactions and/or dynamic covalent bonds into the *in situ*-formed nanodomains to render them robust yet deformable, thereby maximizing energy dissipation while simultaneously ensuring strong interfacial interactions with the surrounding polymer matrix. By increasing the effective cross-linking density, these nanodomains act as nanofillers that improve the tensile strength and modulus of the plastics, while their deformation combined with reversible cross-link dissociation contributes to superior toughness. Moreover, the overall mechanical performance of plastics is governed not only by the degree of mechanical matching between the nanodomains and the surrounding matrix, but also by their characteristic dimensions, uniformity and spatial distribution. Optimizing these parameters is thus critical for achieving plastics that simultaneously exhibit high strength and exceptional toughness. Accordingly, we designed alternate polymers consisting of long, rigid backbones and short, hydrogen-bonded flexible segments, with the entire network further cross-linked through hydrogen bonds. Driven by thermodynamic immiscibility between rigid and flexible chains, the flexible segments self-assemble into phase-separated nanodomains within the hydrogen-bond-cross-linked rigid matrix. These hydrogen-bonded nanodomains feature finely tailored mechanical strength and toughness, together with covalent bonding and strong supramolecular interactions with the matrix. This unique architecture prevents the mechanical deterioration usually caused by flexible chain incorporation, thereby maintaining high strength and modulus while significantly enhancing toughness. To the best of our knowledge, the use of nanodomains that combine mechanical robustness with deformability to toughen plastics has not been previously demonstrated. Following this design principle, we fabricated super-strong and ultra-tough poly(aryl amide)–polyurea (PA–PU) plastics via the copolymerization of long, rigid PA chains with short, soft PU chains. The *in situ* formation of PU-derived nanodomains endowed PA–PU plastics with an outstanding tensile strength of 103.7 MPa, a modulus of 2.5 GPa and a remarkable toughness of 36.1 MJ m^−3^, together with excellent chemical stability and re-processability. Benefiting from their high toughness, PA–PU plastics were further manufactured into high-performance and recyclable carbon fiber (CF)-reinforced composites with excellent low-temperature impact resistance. This versatile strategy enables tunable mechanical properties in strong and tough plastics, paving the way for next-generation sustainable, high-performance polymer materials.

## RESULTS AND DISCUSSION

### Fabrication of reversibly cross-linked PA–PU plastics

**Figure 1.**
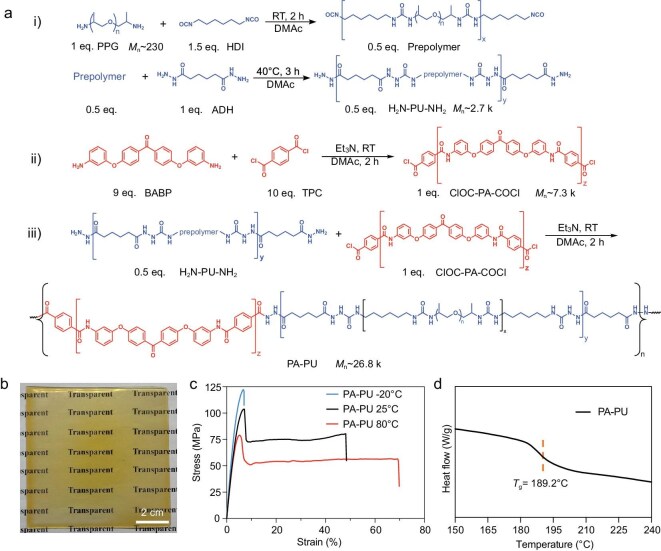
Fabrication and characterization of PA–PU plastics. (a) Synthetic routes for H_2_N-PU-NH_2_ (i), ClOC-PA-COCl (ii) and PA–PU (iii). (b) Digital image of a PA–PU plastic sheet (8 × 8 cm^2^, thickness 0.18 mm). (c) Stress–strain curves of PA–PU plastics measured at different temperatures. (d) DSC curve of PA–PU plastics.

The amino-terminated polyurea (denoted as H_2_N-PU-NH_2_) was synthesized through a two-step process: (i) polyaddition of amino-terminated poly(propylene glycol) (PPG) (number-average molecular weight (*M*_n_) ∼ 230) with hexamethylene diisocyanate (HDI) to obtain isocyanate-terminated prepolymers; and (ii) chain extension with adipic dihydrazide (ADH) to produce H_2_N-PU-NH_2_ [Fig. 1a(i)]. In parallel, the acyl chloride-terminated polyamide (denoted as ClOC-PA-COCl) was synthesized via the polycondensation of 4,4′-bis(3-aminophenoxy) benzophenone (BABP) with terephthaloyl chloride (TPC) [Fig. 1a(ii)]. For H_2_N-PU-NH_2_, the molar ratio of PPG, HMDI and ADH was 1:1.5:1, while for ClOC-PA-COCl, the molar ratio of BABP to TPC was 1:1.11. The successful syntheses of H_2_N-PU-NH_2_ and ClOC-PA-COCl were confirmed by Fourier transform infrared (FT-IR) spectroscopy (Figs S1 and S2). Subsequently, H_2_N-PU-NH_2_ and ClOC-PA-COCl were copolymerized through acyl chloride-amine condensation to yield the PA–PU copolymers [Fig. 1a(iii)]. Detailed procedures for H_2_N-PU-NH_2_, ClOC-PA-COCl and PA–PU are provided in the Supplementary data. The successful synthesis of PA–PU was confirmed by FT-IR spectroscopy (Fig. S3). *M*_n_ values and polydispersity indices (PDIs) of ClOC-PA-COCl, H_2_N-PU-NH_2_ and PA–PU were determined by gel permeation chromatography (GPC) (Table S1). According to the GPC results, PA–PU exhibits an *M*_n_ of 26 847 and a PDI of 1.5. The as-synthesized PA–PU powders were dissolved in *N,N*-dimethylacetamide (DMAc), solution-cast onto glass plates at 60°C, and subsequently heated at 90°C to remove the solvent and obtain PA–PU sheets.

As shown in Fig. 1b, the resulting PA–PU plastic sheet is yellow, transparent and uniform, with dimensions of 8 × 8 cm^2^ and a thickness of 0.18 mm. The UV–vis transmittance spectrum shows that the PA–PU plastic sheet with a thickness of 0.18 mm exhibits a transmittance of 71.3% at 550 nm (Fig. S4). The mechanical properties of PA–PU sheets were characterized by tensile tests at different temperatures with a stretching speed of 50 mm min^−1^ (Fig. 1c and Table S2). At 25°C, the stress–strain curve exhibits the typical features of mechanically tough plastics, including a pronounced cold-drawing plateau after yielding. Under these conditions, PA–PU plastic shows a superhigh yield strength of 103.7 MPa, a Young’s modulus of 2.5 GPa, a strain at break of 48.5%, and an extremely high toughness of 36.1 MJ m^−3^. At −20°C, although chain mobility is largely restricted, PA–PU plastic exhibits mechanical performance comparable to that of conventional engineering plastics at room temperature, with a tensile strength of 122.5 MPa, a higher modulus of 3.3 GPa, and a reduced strain at break of 7.1%. Even at an elevated temperature of 80°C, PA–PU plastic retains a high yield strength of 79.0 MPa and a Young’s modulus of 2.4 GPa, while the strain at break increases to 69.9% and the toughness rises to 39.2 MJ m^−3^. The mechanical properties of PA–PU can also be tailored by varying the molecular weight of the PA segments (Fig. S5). The thermal properties of PA–PU plastics were characterized by thermogravimetric analysis (TGA), differential scanning calorimetry (DSC, Fig. 1d) and dynamic mechanical analysis (DMA). As shown in Fig. S6, the decomposition temperature (*T*_d_), defined as the temperature at 5% weight loss, is 443.0°C, indicating the excellent thermal stability of PA–PU plastic. DSC analysis in Fig. 1d reveals a high glass transition temperature (*T*_g_) of 189.2°C. The DMA curves (Fig. S7) show a high storage modulus of 3.4 GPa at 40°C and 1.9 GPa at 160°C, demonstrating the strong load-bearing capability of PA–PU plastic, even at elevated temperatures. Moreover, both the modulus-temperature and tan δ-temperature curves indicate a sharp decrease in storage modulus above 190.0°C, which closely matches the *T*_g_ determined by DSC.

### Mechanism of high strength and toughness in PA–PU plastics

**Figure 2.**
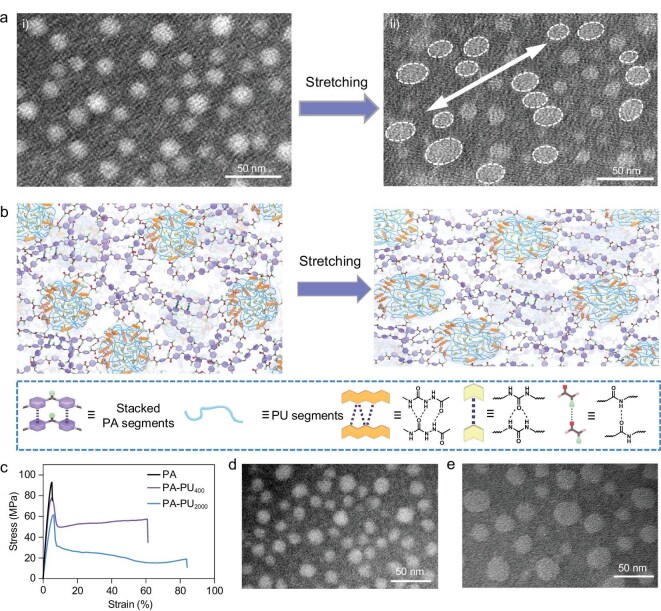
Mechanism of the high strength and toughness of PA–PU plastics. (a) TEM images of the original (i) and stretched (ii) PA–PU plastics. (b) Schematic of the energy dissipation process in PA–PU plastics under stretching. (c) Stress–strain curves of PA, PA–PU_400_ and PA–PU_2000_ plastics. (d and e) TEM images of PA–PU_400_ (d) and PA–PU_2000_ (e) plastics.

The structure of the PA–PU plastics was characterized by transmission electron microscopy (TEM). Before TEM tests, the specimens were stained by sodium phosphotungstate, which preferentially stains the amide groups within the rigid PA segments. As shown in Fig. 2a(i), the TEM image of PA–PU plastic clearly reveals *in situ*-formed phase-separated nanodomains. These nanodomains, with an average diameter of 17.3 ± 1.5 nm, are uniformly dispersed within the plastic. In the PU segments, urea groups form double hydrogen bonds, whereas acylsemicarbazide (ASCZ) units derived from the isocyanate–hydrazide reaction form multiple hydrogen-bond arrays [40–42]. The nanodomains are formed by the aggregation of hydrogen-bond-cross-linked PU chains, whose thermodynamic immiscibility with the rigid PA matrix drives phase separation. Since these *in situ*-formed nanodomains consist of flexible PU segments reversibly cross-linked by hydrogen bonds, they are robust yet deformable under applied stress. To validate this, PA–PU plastic was stretched to fracture and subsequently examined by TEM. As shown in Fig. 2a(ii), the initially spherical nanodomains were elongated into ellipsoidal shapes along the stretching direction, confirming the deformation and alignment of the PU-derived nanodomains. As illustrated in Fig. 2b, stretching induces the gradual dissociation of hydrogen bonds within the nanodomains, enabling their deformation and facilitating chain realignment. This progressive bond dissociation and chain orientation stabilize plastic flow, giving rise to the pronounced cold-drawing plateau observed after yielding and enabling efficient energy dissipation. Moreover, these nanodomains, which are connected to the rigid PA matrix through covalent bonds and hydrogen-bonding interactions, function as physical cross-links to reinforce the tensile strength of PA–PU plastics. Consequently, the mechanically robust yet deformable hydrogen-bond-cross-linked nanodomains are pivotal to the simultaneously enhanced tensile strength, modulus and toughness of PA–PU plastics. For comparison, PA with an *M*_n_ of 26 847 was synthesized (synthetic details are provided in the Supplementary data). No phase-separated nanodomains are observed in the TEM image of the PA sample (Fig. S8). As shown in Fig. 2c, the stress–strain curve of PA exhibits brittle behavior, with a Young’s modulus of 2.6 GPa comparable to that of PA–PU plastic, but a much lower tensile strength (93.3 MPa) and drastically reduced toughness (0.3 MJ m^−3^). These results confirm that the incorporation of robust yet deformable nanodomains in PA–PU plastic significantly enhances tensile strength and toughness without sacrificing modulus.

The size and intrinsic mechanical performance of robust yet deformable nanodomains within the PA matrix play a decisive role in determining the overall mechanical properties of PA–PU plastics. To investigate this, two control PA–PU plastics were synthesized using PPG with *M*_n_ values of 400 and 2000, denoted as PA–PU_400_ and PA–PU_2000_, respectively. The synthetic procedures of PA–PU_400_ and PA–PU_2000_ are identical to those of PA–PU and are provided in the Supplementary data, while their molecular weights were determined by GPC (Table S3). TEM images (Fig. 2d and e) reveal that both PA–PU_400_ and PA–PU_2000_ exhibit phase-separated nanodomains, with average diameters of 18.3 ± 1.6 and 25.0 ± 5.6 nm, respectively. Notably, the nanodomain size increases with increasing PPG molecular weight. Upon stretching to fracture, the nanodomains in both samples elongate along the stretching direction (Fig. S9), revealing their robust yet deformable nature. As shown in Fig. 2c and Table S4, PA–PU_400_ exhibits a yield strength of 77.6 MPa, a Young’s modulus of 2.3 GPa, a strain at break of 61.2% and a toughness of 33.1 MJ m^−3^. In contrast, PA–PU_2000_ shows a lower yield strength (61.8 MPa) and Young’s modulus (1.4 GPa), but a higher elongation at break (84.1%) accompanied by a reduced toughness (19.2 MJ m^−3^). These distinct mechanical properties are attributed to differences in nanodomains; compared with PA–PU, both PA–PU_400_ and PA–PU_2000_ contain longer PU chains, as well as larger and more polydisperse nanodomains. As nanodomains increase in size and polydispersity, the interfacial area per unit volume between the nanodomains and the matrix decreases, thereby weakening stress transfer across the rigid–soft interface and ultimately lowering tensile strength and modulus. Conversely, the enlarged nanodomains are able to accommodate greater deformation, thereby enhancing the strain at break. These results suggest that robust yet deformable nanodomains provide a general strategy for designing mechanically super-strong and tough polymers.

### Chemical stability of PA–PU plastics

**Figure 3.**
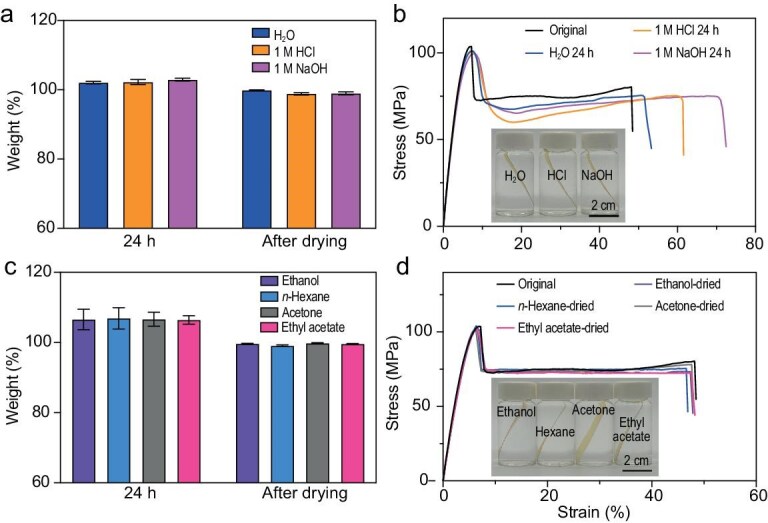
Solvent resistance of PA–PU plastics. (a) Weights of PA–PU plastics after immersion in water, 1 M HCl and 1 M NaOH aqueous solutions for 24 h, followed by drying. (b) Stress–strain curves of PA–PU plastics after immersion in different aqueous solutions for 24 h without drying. Insets show digital images of the immersed samples. (c) Weights of PA–PU plastics after immersion in ethanol, acetone, *n*-hexane and ethyl acetate for 24 h, followed by drying. (d) Stress–strain curves of PA–PU plastics after immersion in various organic solvents for 24 h and subsequent drying at 60°C. Insets show digital images of the immersed samples.

The chemical stability of the PA–PU plastics was evaluated by examining their resistance to aqueous solutions and various organic solvents. Water resistance tests were conducted by immersing plastic sheets (5 cm × 0.5 cm × 0.18 mm) in purified water, 1 M HCl aqueous solution and 1 M NaOH aqueous solution. After 24 h of immersion, the mass gains were ∼2.1%, ∼2.2% and ∼2.9%, respectively (Fig. 3a). The immersed specimens were immediately subjected to tensile tests without drying. As shown in Fig. 3b, the soaked plastic sheets exhibit only a slight decrease in tensile strength (97.7–101.3 MPa) and Young’s modulus (2.3–2.5 GPa), along with an increase in strain at break (53.3%–72.5%). After drying the immersed specimens at 60°C, both the mass and mechanical properties of the PA–PU plastics are almost fully restored (Fig. 3a and Fig. S10). After prolonged immersion in 1 M HCl and 1 M NaOH aqueous solutions, the PA–PU plastics exhibited a slight reduction in tensile strength (Fig. S11). The remarkable water stability of PA–PU plastics arises from their unique structural features, specifically the confinement of hydrogen-bond-cross-linked nanodomains within the hydrophobic microenvironment created by rigid aromatic PA segments. As a result, only a minimal amount of water can penetrate the plastic, partially disrupting the hydrogen bonds and acting as a plasticizer, which slightly decreases the yield strength while increasing the strain at break. The resistance to organic solvents was evaluated by immersing the plastic sheets in ethanol, *n*-hexane, acetone and ethyl acetate for 24 h. The samples remain insoluble, with mass gains of 6.4%–6.9% (Fig. 3c). The soaked specimens exhibited markedly reduced tensile strength and modulus when tested without drying, which can be attributed to solvent-induced swelling of the plastics (Fig. S12). After drying at 60°C, the specimens nearly regain their original weights. As shown in Fig. 3d, the stress–strain curves of the dried specimens closely match that of the pristine sample, demonstrating the excellent chemical stability of PA–PU plastics in the tested organic solvents.

### Fabrication and impact resistance of PA–PU/CF composites

**Figure 4.**
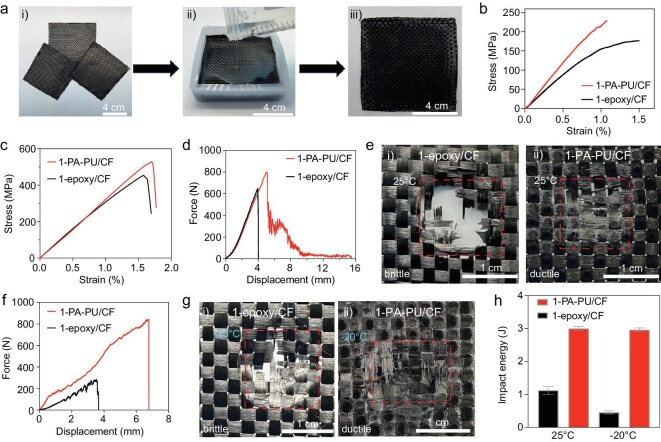
Fabrication and impact resistance of PA–PU/CF composites. (a) Fabrication process of PA–PU/CF composites. (b and c) Flexural (b) and tensile (c) stress–strain curves of 1-PA–PU/CF and 1-epoxy/CF composites. (d) Force–displacement curves of 1-PA–PU/CF and 1-epoxy/CF composites at 25°C. (e) Digital images of fractured 1-epoxy/CF (i) and 1-PA–PU/CF (ii) composites after impact at 25°C. (f) Force–displacement curves of 1-PA–PU/CF and 1-epoxy/CF composites at −20°C. (g) Digital images of fractured 1-epoxy/CF (i) and 1-PA–PU/CF (ii) composites after impact at −20°C. (h) Comparison of impact energies of 1-PA–PU/CF and 1-epoxy/CF composites at 25 and −20°C.

Reversibly cross-linked PA–PU plastics with exceptionally high tensile strength and significantly improved toughness were further employed to fabricate high-performance CF-reinforced polymer composites featuring low-temperature impact resistance and recyclability. As shown in Fig. 4a, three pieces of desized CF cloths were first placed at the bottom of a silicone mold, followed by pouring a DMAc solution of PA–PU (100 mg/mL) [Fig. 4a(i) and (ii)]. After solvent evaporation at 80°C, the resulting pre-impregnated materials (prepregs) were hot-pressed at 140°C for 30 min to obtain sheet-like PA–PU/CF composites [Fig. 4a(iii)]. For comparison, epoxy/CF composites were also fabricated by impregnating CF cloths with commercially available epoxy thermosets (synthetic procedures are provided in the Supplementary data). For simplicity, these two types of composites are denoted as *n*-PA–PU/CF and *n*-epoxy/CF, where *n* represents the number of CF cloth layers. The mass ratio of the CF cloths was fixed at 70% in both of the two types of composites. Figure 4b shows the flexural stress–strain curves of the PA–PU/CF composites. Specifically, the 1-PA–PU/CF composite achieves a flexural strength of 228.9 MPa and a flexural modulus of 25.3 GPa, significantly higher than those of the 1-epoxy/CF composite (175.9 MPa and 14.5 GPa) (Table S5). Consistent with the three-point bending tests, tensile tests further demonstrate the superior mechanical performance of the PA–PU/CF composites. As shown in Fig. 4c, the 1-PA–PU/CF composite exhibits a tensile strength of 529.2 MPa and a Young’s modulus of 33.6 GPa, both surpassing those of the 1-epoxy/CF composite (454.1 MPa and 31.9 GPa; detailed data are summarized in Table S5). As shown in Fig. S13, the 2- and 3-PA–PU/CF composites exhibit mechanical properties comparable to those of the 1-PA–PU/CF composites owing to the fixed CF mass fraction, and a similar trend is observed in the *n*-epoxy/CF composites. The remarkable tensile strength and modulus of the PA–PU/CF composites stem from the mechanically robust and tough PA–PU matrix and the strong interfacial adhesion between PA–PU and CF cloths. The interfacial adhesion strength between an individual CF bundle and the PA–PU matrix was measured to be 17.0 MPa (Fig. S14). Scanning electron microscopy (SEM) images further confirm that the PA–PU plastics fully infiltrated the interstitial regions within the CF bundles (Fig. S15). Upon desizing, the abundant oxygen-containing groups on CF surfaces form robust hydrogen bonds with amide and urea groups in PA–PU plastic [43,44]. In addition, the aromatic PA segments engage in strong π–π stacking interactions with the graphene-like crystalline planes of CF [45,46]. The synergistic contributions of hydrogen bonding and π–π stacking significantly reinforce interfacial adhesion, thereby enabling the fabrication of high-strength PA–PU/CF composites [46,47].

The impact resistance of the PA–PU/CF composites was investigated using a drop-hammer impact tester equipped with an environmental chamber. The maximum impact resistance force (MIRF) and the impact energy at penetration were determined from the force-displacement curves. Figure 4d presents the force–displacement curves of the 1-PA–PU/CF and 1-epoxy/CF composites tested at 25°C. The 1-PA–PU/CF composite exhibits an MIRF of 801.1 N and an impact energy of 3.0 J, both substantially higher than those of the 1-epoxy/CF composite (650.7 N and 1.1 J, respectively). As shown in Fig. 4e(i), the 1-epoxy/CF composite undergoes brittle fracture, characterized by extensive crack propagation and fiber breakage in the impact zone. In contrast, the impacted 1-PA–PU/CF composite displays a ductile fracture with a limited damaged area [Fig. 4e(ii)], demonstrating its superior ability to dissipate impact energy. Figure 4f shows the force–displacement curves of the 1-PA–PU/CF and 1-epoxy/CF composites at −20°C. The 1-PA–PU/CF composite maintains excellent impact resistance, with the MIRF slightly increasing to 840.2 N and the impact energy marginally decreasing to 2.9 J. In sharp contrast, the MIRF and impact energy of the 1-epoxy/CF composite drops drastically to 283.1 N and 0.5 J, respectively. After impact at −20°C, the 1-epoxy/CF composites again exhibit brittle fracture with extensive fiber fragmentation [Fig. 4g(i)], whereas the 1-PA–PU/CF composite retains a ductile fracture mode with limited damage spread [Fig. 4g(ii)], confirming their superior low-temperature impact resistance. As summarized in Fig. 4h, the PA–PU/CF composites display nearly identical impact energies at 25°C and −20°C, while the epoxy/CF composites show a pronouncedly decreased impact energy at low temperature. This highlights the outstanding impact resistance of PA–PU/CF composites, particularly under cryogenic conditions. The superior impact resistance of PA–PU/CF composites arises from the excellent energy-dissipating capacity of the phase-separated nanodomains within PA–PU. Even at low temperatures, the hydrogen bonds can dissociate and the nanodomains undergo deformation, thereby effectively dissipating impact energy. By contrast, the stress–strain curves of epoxy resin at both room and low temperatures (Fig. S16) exhibit inherently brittle behavior. At 25°C, the tensile strength, Young’s modulus and toughness are 86.6 MPa, 1.9 GPa and 0.32 MJ m^−3^, respectively. These values change to 101.6 MPa, 3.2 GPa and 0.33 MJ m^−3^ at −20°C, respectively, accounting for the inferior impact resistance of the epoxy/CF composites.

### Re-processability of PA–PU plastics and recyclability of PA–PU/CF composites

**Figure 5.**
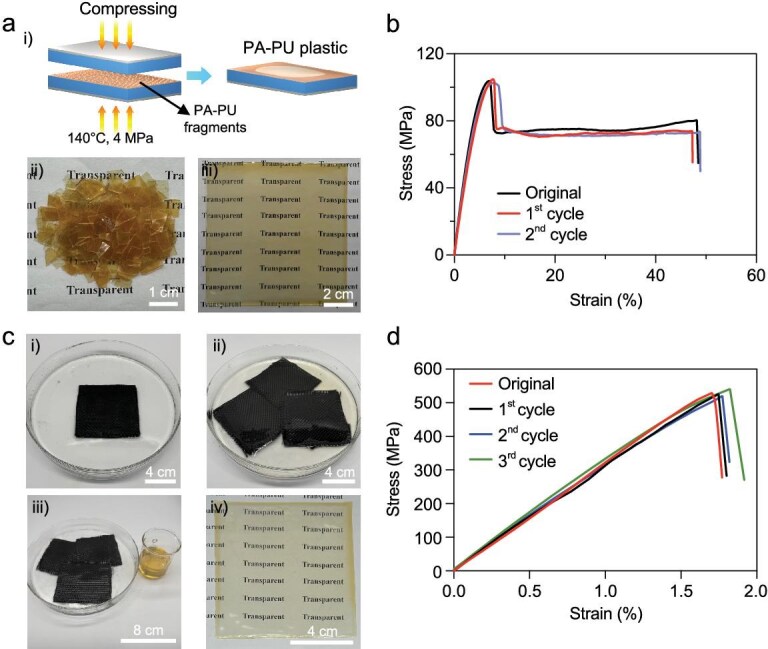
Re-processing of PA–PU plastics and recycling of PA–PU/CF composites. (a) Schematic illustration (i) and digital images (ii and iii) of the re-processing procedure. (b) Stress–strain curves of the original and re-processed PA–PU plastics after the first and second recycling cycles. (c) Digital images of the recycling procedure for PA–PU/CF composites. (d) Stress–strain curves of the original and recycled PA–PU/CF composites obtained from recovered CF cloths and PA–PU.

Because the PA–PU plastics are reversibly cross-linked with hydrogen bonds, they can be readily re-processed upon heating in the presence of trace amounts of polar solvents. The schematic illustration of the re-processing procedure is shown in Fig. 5a(i). As shown in Fig. 5a(ii), PA–PU sheets were cut into millimeter-sized pieces, treated with two drops of DMAc, and hot-pressed at 140°C for 30 min under 4 MPa. Subsequently, the fragments were successfully consolidated into an intact sheet [Fig. 5a(iii)]. As depicted in Fig. 5b, the stress–strain curve of the reprocessed PA–PU sheet closely overlaps with that of the pristine one, confirming its excellent re-processability. During reprocessing, DMAc swells the polymer chains and partially disrupts hydrogen bonds, thereby enhancing chain mobility at elevated temperatures and enabling bond reorganization. This process was repeated twice without any loss of mechanical performance.

Moreover, because PA–PU is soluble in DMAc, the PA–PU/CF composites exhibit solvent-assisted recyclability. As shown in Fig. 5c(i), 3-PA–PU/CF composites were immersed in 30 mL of DMAc. After 6 h of immersion at room temperature, the PA–PU plastics dissolved [Fig. 5c(ii)], and the CF cloths were readily separated from the solution. The cloths were then thoroughly washed with ethanol to yield clean recovered CF cloths [Fig. 5c(iii)], while the DMAc solution was cast onto a glass plate to obtain recovered PA–PU plastics [Fig. 5c(iv)]. SEM images (Fig. S17) and Raman spectra (Fig. S18) confirm the non-destructive recovery of CF cloths [48]. Meanwhile, the stress–strain curves and FT-IR spectra of the recovered PA–PU closely match those of the original sample, while the *T*_g_ values remain nearly identical, confirming the high purity of the recovered PA–PU plastics (Fig. S19). Finally, the recovered CF cloths and PA–PU were reused to fabricate new PA–PU/CF composites, which display mechanical properties indistinguishable from those of the original composites, even after three cycles, thereby demonstrating their excellent recyclability (Fig. 5d).

## CONCLUSION

We demonstrate the fabrication of mechanically super-strong and ultra-tough PA–PU plastics by incorporating rigid yet deformable nanodomains with high energy-dissipation capacity into a hydrogen-bonded rigid network. The PA–PU plastics are reversibly cross-linked through hydrogen bonds and *in situ*-formed PU nanodomains. These nanodomains endow the plastics with an exceptional yield strength of 103.7 MPa, a high Young’s modulus of 2.5 GPa and an extraordinary toughness of 36.1 MJ m^−3^. Since the hydrogen-bonded nanodomains are confined within the hydrophobic and aromatic PA matrix, the PA–PU plastics display remarkable thermal stability and solvent resistance. Benefiting from their outstanding strength and toughness, PA–PU/CF composites surpass conventional epoxy/CF composites in both mechanical strength and low-temperature impact resistance. Furthermore, the dynamic nature of hydrogen bonds and nanodomains ensures re-processability of PA–PU plastics and recyclability of PA–PU/CF composites. Robust yet deformable nanodomains thus provide a unique strategy to simultaneously strengthen and toughen plastics. Anchored to the rigid matrix via covalent and supramolecular bonds, they efficiently transfer stress while dissipating energy through reversible bond dissociation and nanodomain deformation, thereby eliminating the long-standing trade-off among strength, modulus and toughness. Importantly, their reversible nature also preserves recyclability and re-processability, rendering this design highly promising for lightweight and sustainable polymer materials in aerospace, automotive and other demanding engineering applications.

## MATERIALS AND METHODS

All experimental details and methods are provided in the Supplementary data.

## Supplementary Material

nwaf512_Supplemental_File
