# Supplementary material for: Robust yet deformable nanodomains for super-strong and ultra-tough reversibly cross-linked plastics
Source: Natl Sci Rev. 2025 Nov 17;12(12):nwaf512. doi: 10.1093/nsr/nwaf512 (PMC12707060; doi:10.1093/nsr/nwaf512)
Supplement: nwaf512_Supplemental_File [file nwaf512_supplemental_file.pdf]

Supplementary information

Robust yet Deformable Nanodomains for Super-Strong and Ultra-Tough Reversibly Cross-linked Plastics

*Chengliang Tao, Xingyuan Lu, Xiang Li and Junqi Sun**

State Key Laboratory of Supramolecular Structure and Materials, College of Chemistry, Jilin University, Changchun 130012, P. R. China

E-mail: sun_junqi@jlu.edu.cn

Tel: 86-431-85168723

Materials. 4,4'-Bis(3-aminophenoxy) benzophenone (BABP) and amino-terminated polypropylene glycol (PPG, number-average molecular weight (M_n) ≈ 230, 400 and 2,000) were purchased from Macklin. Prior to polymerization, the BABP powders were washed with water to remove impurities. Terephthaloyl chloride (TPC), 4,4'-methylenedianiline (MDA), and adipic dihydrazide (ADH) were obtained from Tokyo Chemical Industry. *N*, *N*-Dimethylacetamide (DMAc) and triethylamine (TEA) were purchased from Innochem. Hexamethylene diisocyanate (HDI) was purchased from Sigma-Aldrich. 2,2-Bis(4-glycidyloxyphenyl)propane was obtained from Dow Chemical Company. The carbon fiber (CF) cloths were obtained from Toray T300. The CF cloths were first incinerated in air and then soaked in ethanol at 50 ℃ for 24 h. After washing with distilled water and drying at 50 ℃, the CF cloths were employed for the fabrication of CF-reinforced polymer composites.

Characterization. Fourier transform infrared (FT-IR) spectra were recorded on a Bruker VERTEX 80 V FT-IR spectrometer. UV-vis transmittance spectrum was conducted by a PerkinElmer Lambda 365 spectrometer. Raman spectra were conducted using a LabRAM ARAMIS Smart Raman Spectrometer with the excitation wavelength of 633 nm. Stress-strain curves were obtained using a universal testing machine (Instron 68TM-5 Tension Instrument). Molecular weights and polydispersity indices (PDIs) of the polymers were determined by gel permeation chromatography (GPC, Waters 1515) with *N*, *N*-dimethylformamide (DMF) as the eluent. Thermal stability analysis of the plastics was conducted using a TA Instruments Q500

thermogravimetric analyzer. Samples (5 mg) was heated from 30 to 800 ℃ at a rate of 10 ℃/min under nitrogen flow. Glass transition temperatures (T_g) were measured using a TA Instruments Q200 differential scanning calorimeter. Samples (5 mg) were heated from 30 to 300 ℃ at 10 ℃/min under nitrogen flow. Dynamic mechanical analysis (DMA) was performed on a TA Instruments Q800 in tensile mode, with a strain of 0.1%, a frequency of 1 Hz, and a heating rate of 5 °C/min. Transmission electron microscope (TEM) images were recorded using JEM-2100 microscope operating at an acceleration voltage of 200 kV. Samples were negatively stained with sodium phosphotungstate prior to TEM observation. Scanning electron microscopy (SEM) images were obtained using an XL30 ESEM FEG scanning electron microscope. Impact resistance under varying impact velocities was tested using a drop-hammer impact tester (Instron CEAST 9440HV). A hemispherical impactor with a diameter of 12.7 mm and a mass of 1.0 kg was released from different heights. The lower fixture consisted of a holder with an outer diameter of 60 mm and an inner diameter of 40 mm, while the upper fixture was a press board with a 40 mm-diameter circular hole at its center. All specimens for impact-resistance tests were prepared with dimensions of 65 × 65 × 0.23 mm^3. The maximum impact resistance force (MIRF) and impact energy at penetration were determined from complete sample penetration by the drop hammer [1].

Synthesis of ClOC-PA-COCl. BABP (3.57 g, 9 mmol) and TEA (4.2 mL, 30 mmol) were dissolved in anhydrous DMAc (36 mL) in a 100 mL round-bottom flask. A solution of TPC (2.03 g, 10 mmol) in DMAc (20 mL) was then added dropwise to the above mixture. The reaction mixture was stirred at room temperature for 2 h, and the resulting solution was directly used for subsequent polymerization without further purification.

Synthesis of H_2N-PU-NH_2. PPG ($M_n \approx 230$, 0.23 g, 1 mmol) was dissolved in DMAc (3 mL) in a 50 mL round-bottom flask. A solution of HDI (0.252 g, 1.5 mmol) in DMAc (3 mL) was added dropwise into the flask, and the mixture was stirred for 2 h to form the prepolymer. Subsequently, a solution of ADH (0.174 g, 1 mmol) in DMAc (10 mL) was added, and the reaction was continued for 3 h at 40 ℃ to yield

H_2N-PU-NH_2. The syntheses of H_2N-PU_{400}-NH_2 and H_2N-PU_{2000}-NH_2 followed the same procedure of H_2N-PU-NH_2, except that PPG ($M_n \approx 230$, 0.23 g, 1 mmol) was replaced by PPG ($M_n \approx 400$, 0.40 g, 1 mmol) and PPG ($M_n \approx 2000$, 2.00 g, 1 mmol), respectively.

Preparation of the PA-PU plastics. The DMAc solution of H_2N-PU-NH_2 was added dropwise to the DMAc solution of ClOC-PA-COCl, and the mixture was stirred at room temperature for 2 h. The resulting polymer solution was then poured into ethanol (300 mL) to precipitate the product. The precipitates were collected by filtration, washed with water, and dried at 60 ℃ to yield PA-PU copolymers as white powders. The yield of the PA-PU plastics is 92.7%. These powders were dissolved in DMAc and cast onto glass substrates at 60 ℃, followed by heating at 90 ℃ to form sheet-like PA-PU plastics. The preparations of PA-PU_{400} and PA-PU_{2000} followed the same procedure, except that the DMAc solution of H_2N-PU-NH_2 was replaced with H_2N-PU_{400}-NH_2 and H_2N-PU_{2000}-NH_2, respectively.

Preparation of the PA plastics. A solution of TPC (2.10 g, 10 mmol) in DMAc (22 mL) was added dropwise to a DMAc solution (40 mL) of BABP (3.96 g, 10 mmol) and TEA (4.2 mL, 30 mmol). The mixture was stirred at room temperature for 4 h and then poured into ethanol (300 mL) to precipitate the product. The precipitates were collected by filtration, thoroughly washed with water, and dried at 60 ℃ to yield PA powders. These powders were dissolved in DMAc and cast onto glass substrates at 60 ℃, followed by heating at 90 ℃ to form PA plastic sheets.

Preparation of the PA-PU/CF composites. Using 3-PA-PU/CF as a representative example, the preparation was carried out as follows. A DMAc solution of PA-PU (100 mg/mL) was poured into a silicone mold containing three pieces of CF cloth. The prepregs were heated at 80 ℃ to remove the solvent and subsequently hot-pressed at 140 ℃ for 30 min to obtain sheet-like 3-PA-PU/CF composites.

Preparation of epoxy resins. 2,2-Bis(4-glycidyloxyphenyl)propane (2.06 g, 6 mmol) and MDA (0.64 g, 3 mmol) were dissolved in DMAc (30 mL), and the solution was poured into a silicone mold. The solvent was removed by heating at 80 ℃, affording

the prepolymer, which was subsequently cured at 140 ℃ for 2 h to yield the epoxy resins.

Preparation of epoxy/CF composites. Using 3-epoxy/CF as a representative example, the preparation was carried out as follows. 2,2-Bis(4-glycidyloxyphenyl)propane (1.03 g, 3 mmol) and MDA (0.32 g, 1.5 mmol) were dissolved in DMAc (15 mL), and the solution was poured into a silicone mold containing three pieces of CF cloth. The resulting prepregs were heated at 80 ℃ to remove the solvent and subsequently cured at 140 ℃ for 2 h to yield sheet-like 3-epoxy/CF composites.

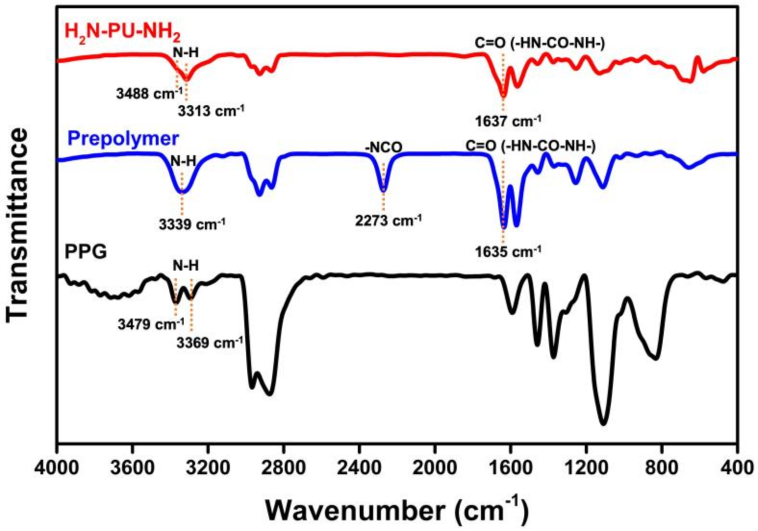

Figure S1. FT-IR spectra of PPG, prepolymer, and H_2N-PU-NH_2.

In the FT-IR spectrum of PPG, the characteristic peaks at 3479 and 3369 cm^{-1} are assigned to the N-H stretching vibrations of amino groups. In the FT-IR spectrum of prepolymers, the peaks at 3339 cm^{-1}, 2273 cm^{-1}, and 1635 cm^{-1} correspond to the N-H stretching of secondary amine in urea, the stretching of isocyanate groups, and the C=O stretching of urea, respectively, confirming the successful formation of the prepolymers. In the spectrum of H_2N-PU-NH_2, the characteristic peak at 2273 cm^{-1} disappears, and the peak of the C=O band shifts from 1635 to 1637 cm^{-1}. Meanwhile, the characteristic peak at 3339 cm^{-1} disappears, while the peaks at 3488 cm^{-1} and 3313 cm^{-1} correspond to the N-H stretching of primary amine. These results verify the successful synthesis of H_2N-PU-NH_2.

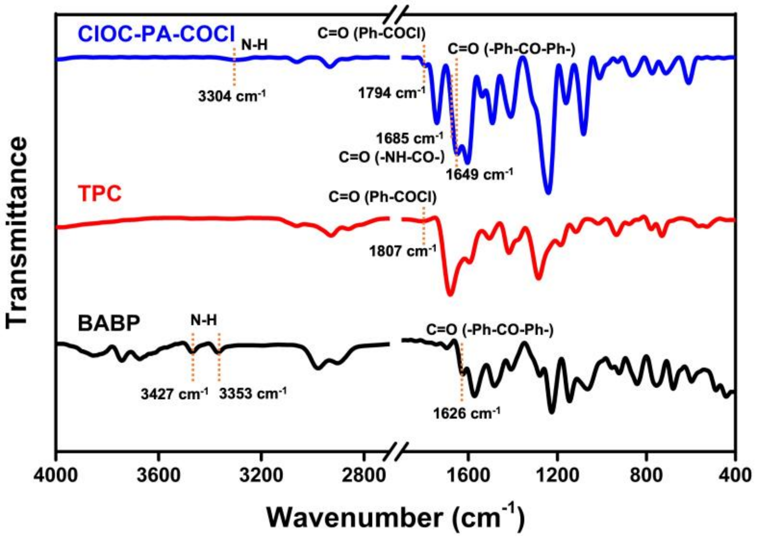

Figure S2. FT-IR spectra of BABP, TPC, and ClOC-PA-COCl.

In the FT-IR spectrum of BABP, the characteristic peaks at 3427 cm^{-1} and 3353 cm^{-1} are attributed to the N-H stretching vibrations of amino groups, while the peak at 1626 cm^{-1} corresponds to the C=O stretching vibration of ketone groups. In the spectrum of TPC, the peak at 1807 cm^{-1} is assigned to the C=O stretching vibration of acyl chloride. After copolymerization of TPC with BABP, the peaks at 3427 cm^{-1} and 3353 cm^{-1} disappear, and a new peak at 3304 cm^{-1} is observed, corresponding to the N-H stretching of secondary amines. Meanwhile, the C=O peak of ketone groups shifts from 1626 cm^{-1} to 1649 cm^{-1}, and the C=O peak of acyl chloride shifts from 1807 cm^{-1} to 1794 cm^{-1}. In addition, a new peak emerges at 1685 cm^{-1}, which is assigned to the C=O stretching vibration of amide groups. These spectral changes confirm the successful synthesis of ClOC-PA-COCl.

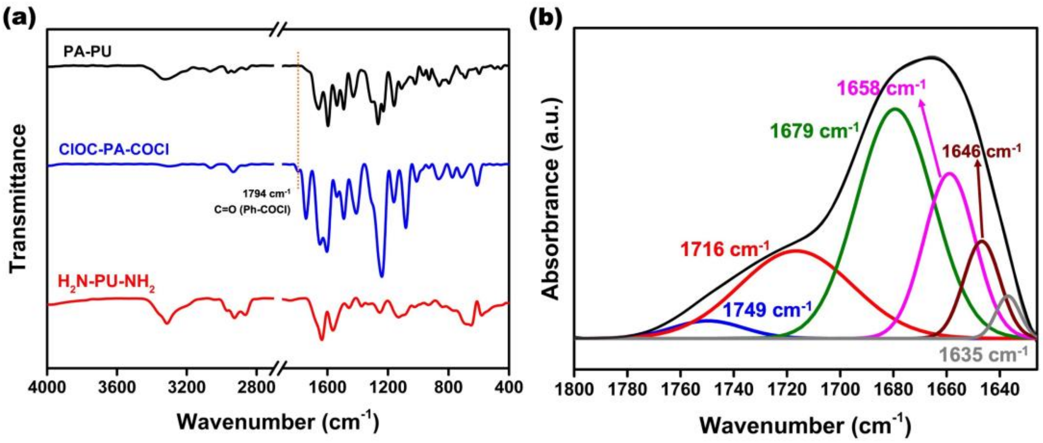

Figure S3. (a) FT-IR spectra of ClOC-PA-COCl, H_2N-PU-NH_2 and PA-PU. (b) Carbonyl (C=O) region of the FT-IR spectrum of the PA-PU plastics with peak deconvolution.

After copolymerization of ClOC-PA-COCl with H_2N-PU-NH_2, the characteristic peak at 1794 cm^{-1}, corresponding to the C=O stretching of acyl chloride, disappears, confirming the successful formation of PA-PU. The carbonyl region of the FT-IR spectrum of PA-PU was deconvoluted (Figure S3b), revealing six fitted peaks at 1749, 1716, 1679, 1658, 1646, and 1636 cm^{-1}. The peaks at 1749 and 1716 cm^{-1} are assigned to the C=O stretching vibrations of acylsemicarbazide groups, while the peak at 1679 cm^{-1} corresponds to the C=O stretching vibrations of amide and free urea groups. The peaks at 1658 and 1646 cm^{-1} are attributed to disordered hydrogen-bonded urea C=O groups, and the peak at 1636 cm^{-1} is associated with ordered hydrogen-bonded urea C=O groups [2,3].

Table S1. M_n, weight-average molecular weights (M_w) and PDI of ClOC-PA-COCl, H_2N-PU-NH_2 and PA-PU.

	M_n	M_w	PDI
ClOC-PA-COCl	7354	10218	1.39
H_2N-PU-NH_2	2712	3380	1.25
PA-PU	26847	39950	1.49

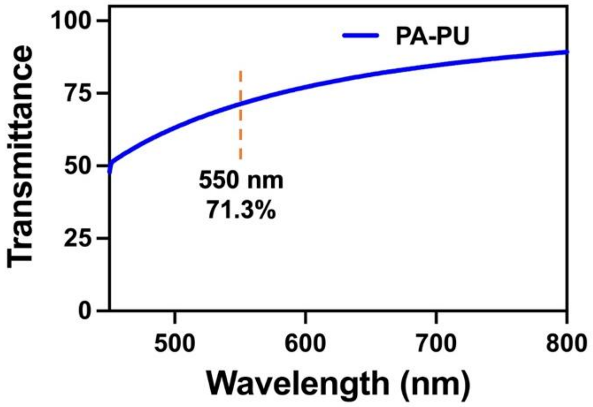

Figure S4. UV-vis transmittance spectrum of the PA-PU plastics with a thickness of 0.18 mm.

Table S2. Mechanical properties of PA-PU plastics measured at different temperatures.

Tested temperatures	Yield strength (MPa)	Young's modulus (GPa)	Strain at break (%)	Toughness (MJ/m^3)
80 ℃	79.0±2.1	2.4±0.1	69.9±2.5	39.2±3.7
25 ℃	103.7±2.3	2.5±0.1	48.5±2.4	36.1±3.0
-20 ℃	122.5±5.1	3.3±0.2	7.1±0.5	0.6±0.1

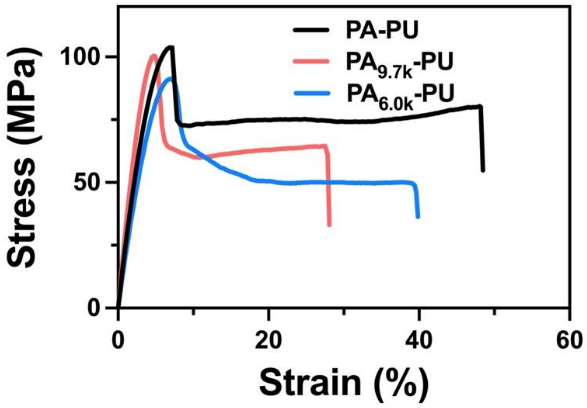

Figure S5. Stress-strain curves of PA-PU, $PA_{9.7k}$-PU and $PA_{6.0k}$-PU.

As shown in Figure S5, two control polymers were prepared using PA segments with M_n of 6.0 k and 9.7 k, while keeping the PU segments identical to those in PA-PU (here PA has a M_n of 7.4 k). For simplicity, these two polymers are denoted as $PA_{6.0k}$-PU and $PA_{9.7k}$-PU, respectively. $PA_{6.0k}$-PU exhibits a tensile strength of 91.2 MPa and a Young's modulus of 2.1 GPa, whereas $PA_{9.7k}$-PU shows a tensile strength of 100.7 MPa and a Young's modulus of 2.9 GPa. With increasing M_n of the PA segments, the Young's modulus gradually increases due to enhanced chain rigidity. In contrast, the tensile strength of $PA_{6.0k}$-PU is lower than that of PA-PU because of the reduced fraction of rigid chains. When M_n increases to 9.7 k, the excessive chain rigidity renders $PA_{9.7k}$-PU relatively brittle, resulting in decreased tensile strength and reduced elongation at break.

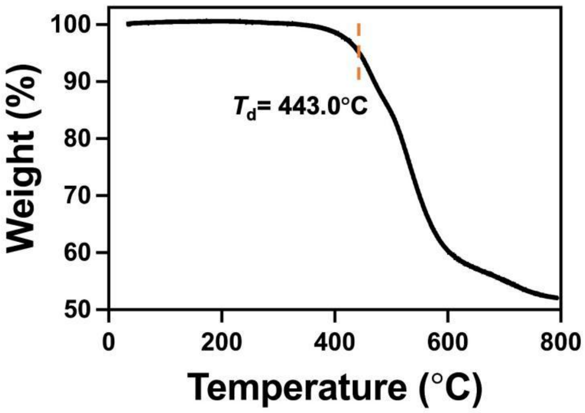

Figure S6. TGA curve of PA-PU plastics.

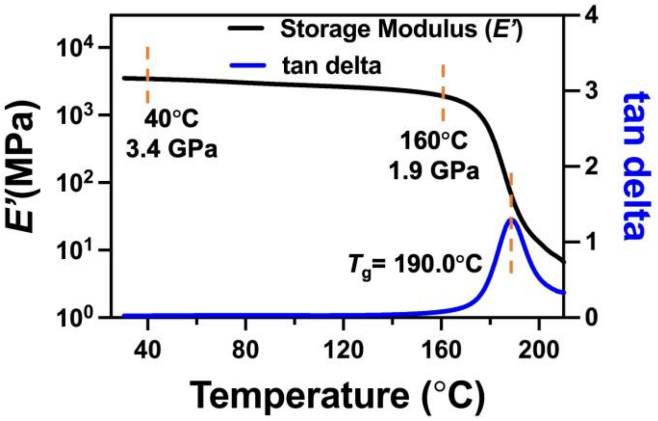

Figure S7. DMA curves of PA-PU plastics.

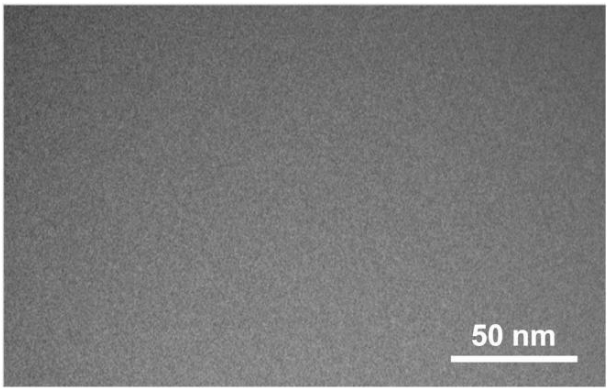

Figure S8. TEM image of PA plastics.

Table S3. M_n, M_w and PDI of PA, H_2N-PU_{400}-NH_2, H_2N-PU_{2000}-NH_2, PA-PU_{400} and PA-PU_{2000}.

	M_n	M_w	PDI
PA	26831	37453	1.40
H_2N-PU_{400}-NH_2	3903	4801	1.23
H_2N-PU_{2000}-NH_2	7095	7886	1.11
PA-PU_{400}	29294	43017	1.47
PA-PU_{2000}	37959	53270	1.40

Table S4. Mechanical properties of PA-PU_{400}, PA-PU_{2000} and PA.

	Yield strength (MPa)	Young's modulus (GPa)	Strain at break (%)	Toughness (MJ/m^3)
PA-PU_{400}	77.6±2.6	2.3±0.1	61.2±1.7	33.1±2.5
PA-PU_{2000}	61.8±3.1	1.4±0.1	84.1±3.0	19.2±0.8
PA	93.3±4.3	2.6±0.2	5.4±1.3	0.3±0.1

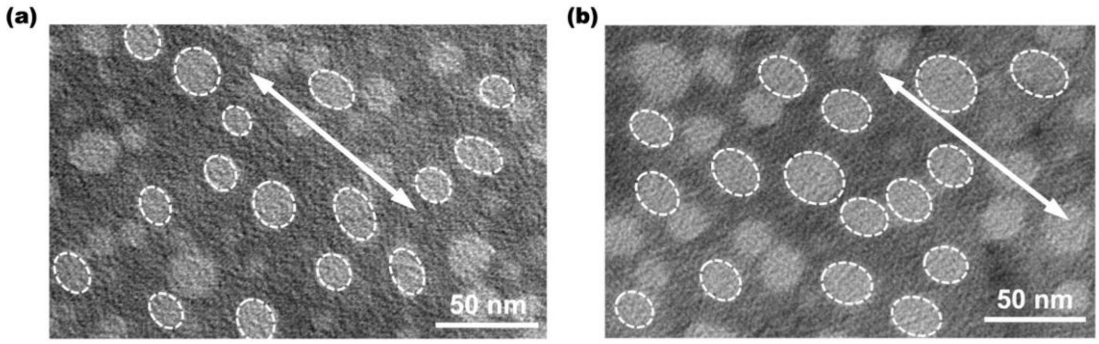

Figure S9. TEM images of the stretched PA-PU_{400} (a) and PA-PU_{2000} (b) plastics.

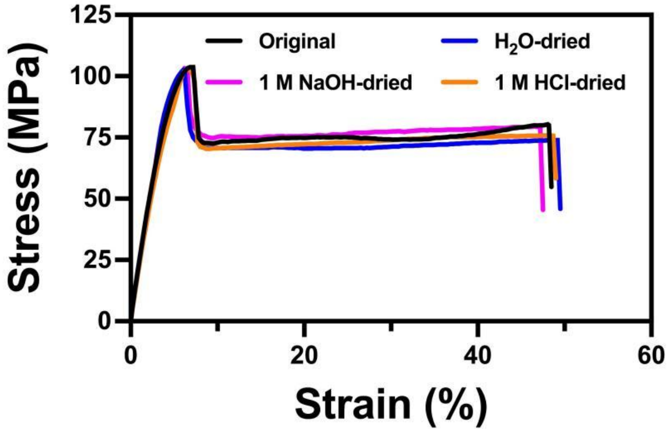

Figure S10. Stress-strain curves of PA-PU plastics after immersion in different aqueous solutions for 24 h and subsequent drying at 60 ℃.

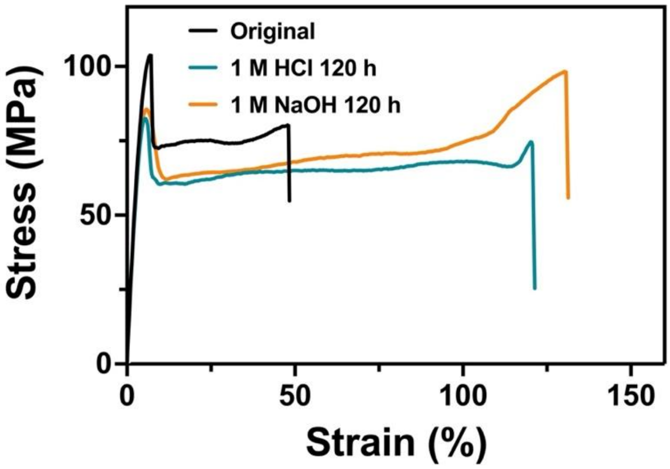

Figure S11. Stress-strain curves of PA-PU plastics immersed in 1 M HCl and 1 M NaOH aqueous solution for 120 h.

As shown in Figure S12, the yield strength decreased from 103.7 MPa to 82.6 MPa after immersion in 1 M HCl and to 85.6 MPa after immersion in 1 M NaOH, while the Young's modulus decreased slightly from 2.5 GPa to 2.3 GPa (1 M HCl) and 2.4 GPa (1 M NaOH). These results demonstrate that the PA-PU plastics retain robust mechanical performance even after prolonged exposure to strong acidic and basic environments.

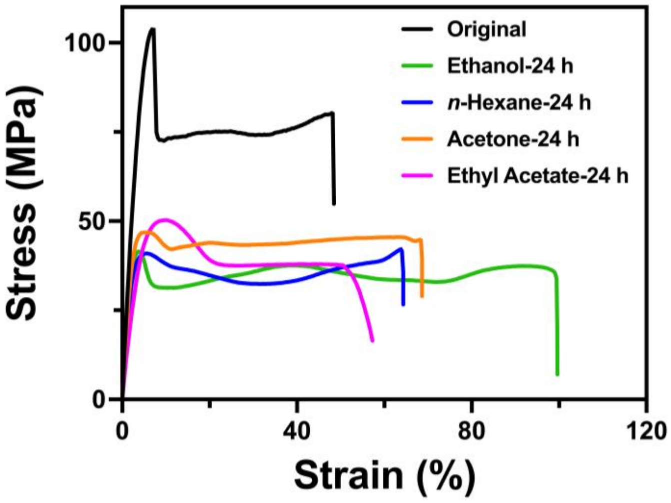

Figure S12. Stress-strain curves of PA-PU plastics after immersion in various organic solvents for 24 h.

Table S5. Mechanical properties of PA-PU/CF composites and epoxy/CF composites.

	Tensile Strength (MPa)	Young's modulus (GPa)	Flexural Strength (MPa)	Flexural modulus (GPa)
PA-PU/CF	529.2±7.8	33.6±0.6	228.9±1.6	25.3±0.7
epoxy/CF	454.1±2.2	31.9±1.1	175.9±2.1	14.5±0.3

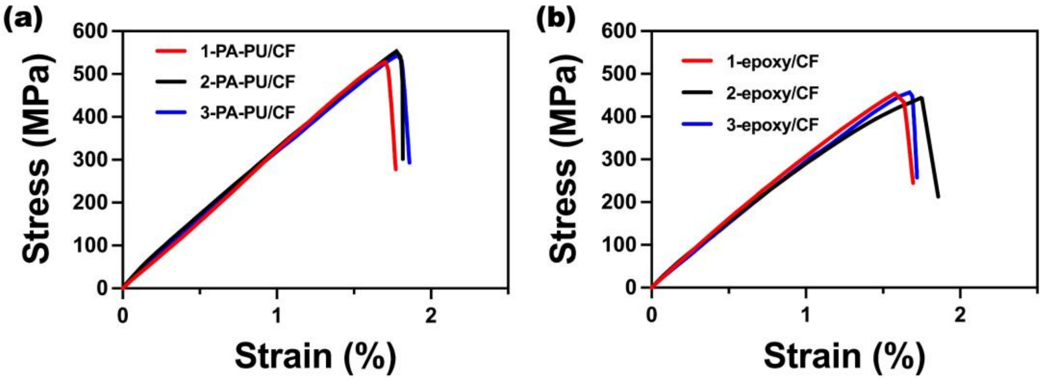

Figure S13. (a, b) Stress-strain curves of PA-PU/CF (a) and epoxy/CF (b) composites with different numbers of CF cloth layers.

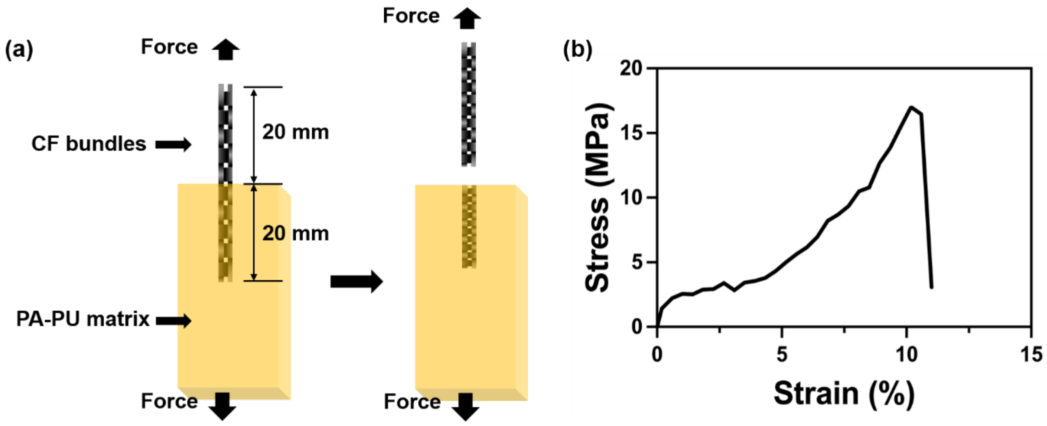

Figure S14. (a) Schematic illustration of the single CF bundle pull-out test. (b) Representative stress-strain curve obtained during the pull-out tests.

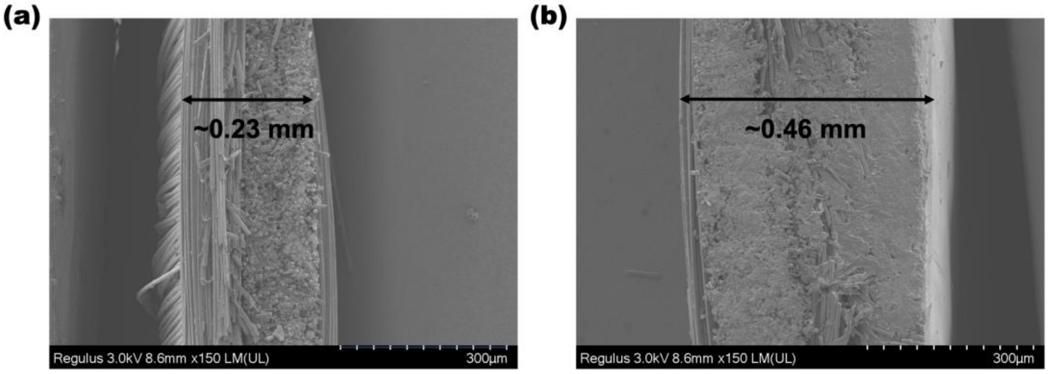

Figure S15. Cross-sectional SEM images of 1-PA-PU/CF (a) and 2-PA-PU/CF (b) composites.

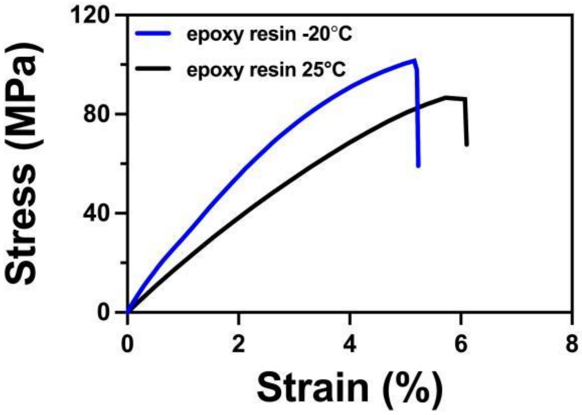

Figure S16. Stress-strain curves of epoxy resins measured at 25 and -20 ℃.

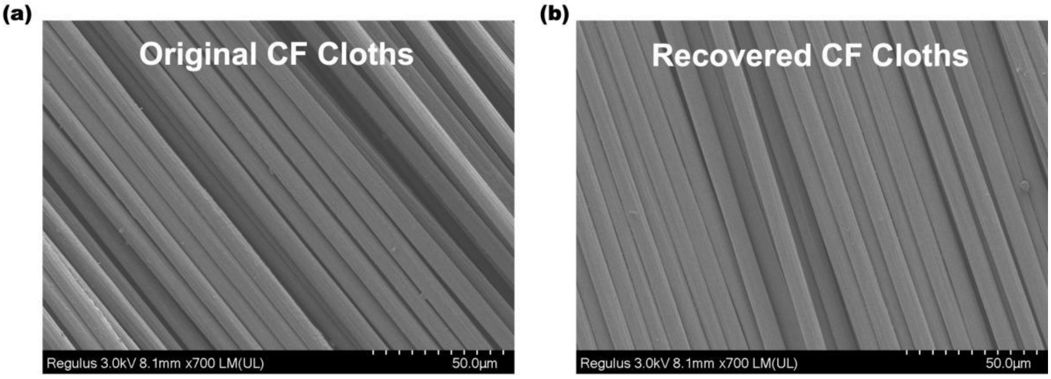

Figure S17. (a, b) SEM images of the original (a) and recovered (b) CF cloths.

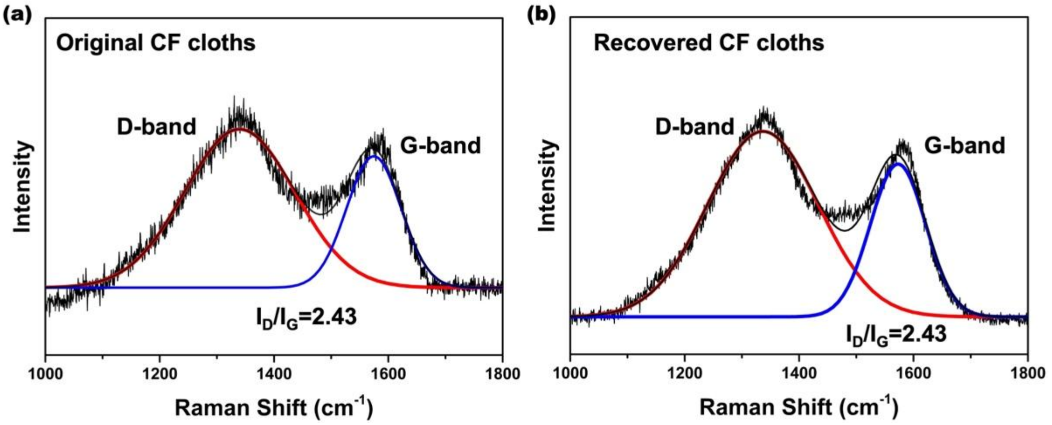

Figure S18. Raman spectra of the original (a) and recovered (b) CF cloths.

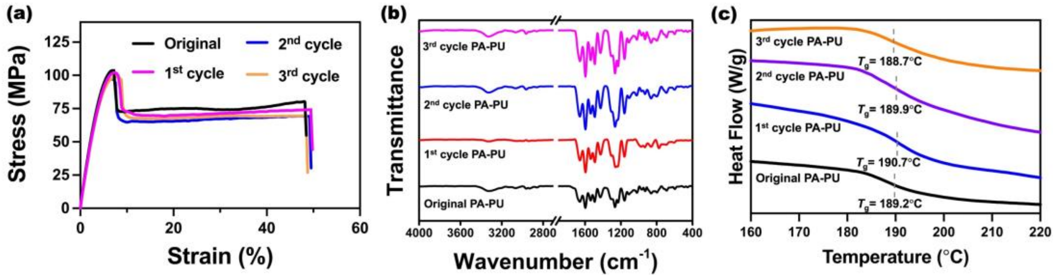

Figure S19. (a-c) Stress-strain curves (a), FT-IR spectra (b) and DSC curves (c) of the original and recovered PA-PU plastics obtained from PA-PU/CF composites.

Reference

1. Wang W, Li Y, Ma Z *et al*. Ultra-Tough Poly(Urea-Urethane) Plastics With Superior Impact Resistance for Cryogenic Applications. *Adv Mater* 2025; **37**: 2509421.

2. Li Z, Zhu Y, Niu W *et al*. Healable and Recyclable Elastomers with Record-High Mechanical Robustness, Unprecedented Crack Tolerance, and Superhigh Elastic Restorability. *Adv Mater* 2021; **33**: 2101498.

3. Guan T, Wang X, Zhu Y *et al*. Mechanically Robust Skin-like Poly(urethane-urea) Elastomers Cross-Linked with Hydrogen-Bond Arrays and Their Application as High-Performance Ultrastretchable Conductors. *Macromolecules* 2022; **55**: 5816-5825.
